# A rare cause of posttraumatic esophageal perforation successfully treated with a new vacuum stent

**DOI:** 10.1055/a-2622-4789

**Published:** 2025-07-09

**Authors:** Marco Magistroni, Giuseppe Iabichino, Milena Di Leo, Monica Arena, Fabrizio Sammartano, Gianfranco Donatelli, Luca De Luca

**Affiliations:** 1 444273 Gastroenterology and Digestive Endoscopy Unit, ASST Santi Paolo e Carlo, Milan, Italy; 2 444273 Trauma Center, San Carlo Borromeo Hospital, ASST Santi Paolo e Carlo, Milan, Italy; 3 55727 Unité dʼEndoscopie Thérapeutique, Hôpital des Peupliers, Paris, France; 4 9307 Departement of Clinical Medicine and Surgery, University of Naples Federico II, Napoli, Italy

Traumatic transmural esophageal defects are a rare condition, recorded in less than 1% of patients managed in trauma centers 1 2 .

We report the case of a 29-year-old male patient who presented to the Emergency Room following a road accident. Abdominal and thoracic computed tomography (CT) revealed a hemoperitoneum with a traumatic splenic injury and a mediastinal collection with pneumomediastinum ( Video 1 and Fig. 1 ). The patient underwent an emergency splenectomy, followed by the placement of mediastinal and thoracic drainages. An intraoperative upper endoscopy was performed, which identified a 3-cm transmural esophageal defect ( Fig. 2 ). An esophageal fully covered metal stent was placed and the patient was subsequently admitted to the intensive care unit. Six days after the stent placement, a new increase in inflammatory markers was noted and a new CT scan showed expansion of the mediastinal collection. Following a multidisciplinary meeting, it was decided to replace the esophageal metal stent with a VacStent (MICRO-TECH Europe GmbH, Dusseldorf, Germany). Three sessions were performed, in which the VacStent was changed every 6–7 days. The defect showed continuous healing progression until it was no longer detectable during endoscopy, as also confirmed by both CT scan and X-ray with an oral contrast agent ( Fig. 3 , Fig. 4 ). On the 39th day after admission to the intensive care unit, the patient was transferred to the surgery department. He was finally discharged in good clinical condition after a total of 57 days of hospitalization. A follow-up upper endoscopy, performed 6 months later, confirmed complete resolution with regular scarring ( Fig. 5 ) and the patient reported eating normally without any symptoms.

VacStent in posttraumatic esophageal perforation.Video 1

**Fig. 1:**
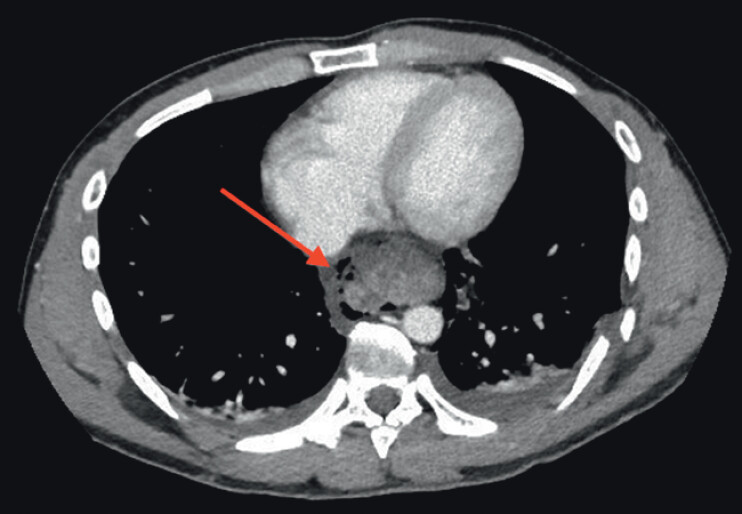
Esophageal perforation on computed tomography scan.

**Fig. 2:**
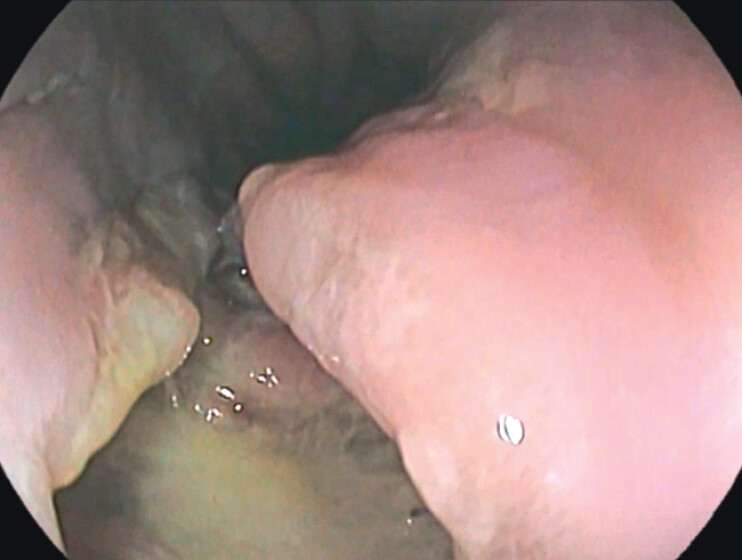
Endoscopic view of esophageal perforation.

**Fig. 3:**
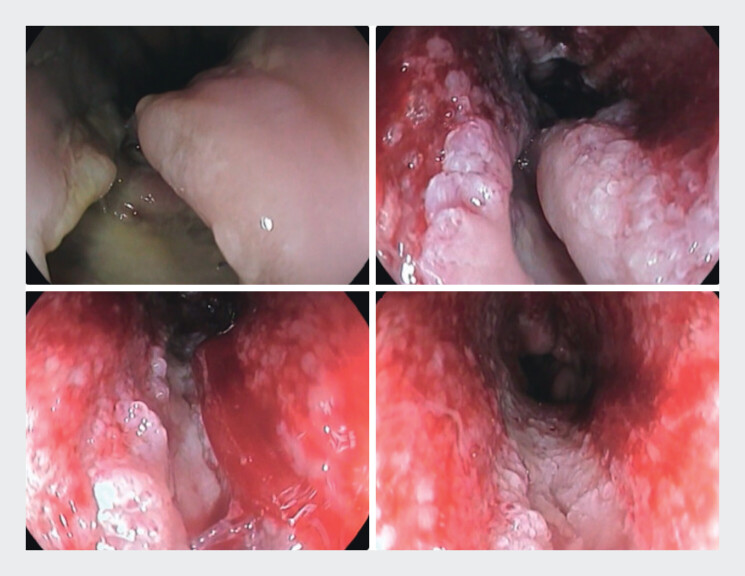
Healing progression after treatment with VacStent.

**Fig. 4:**
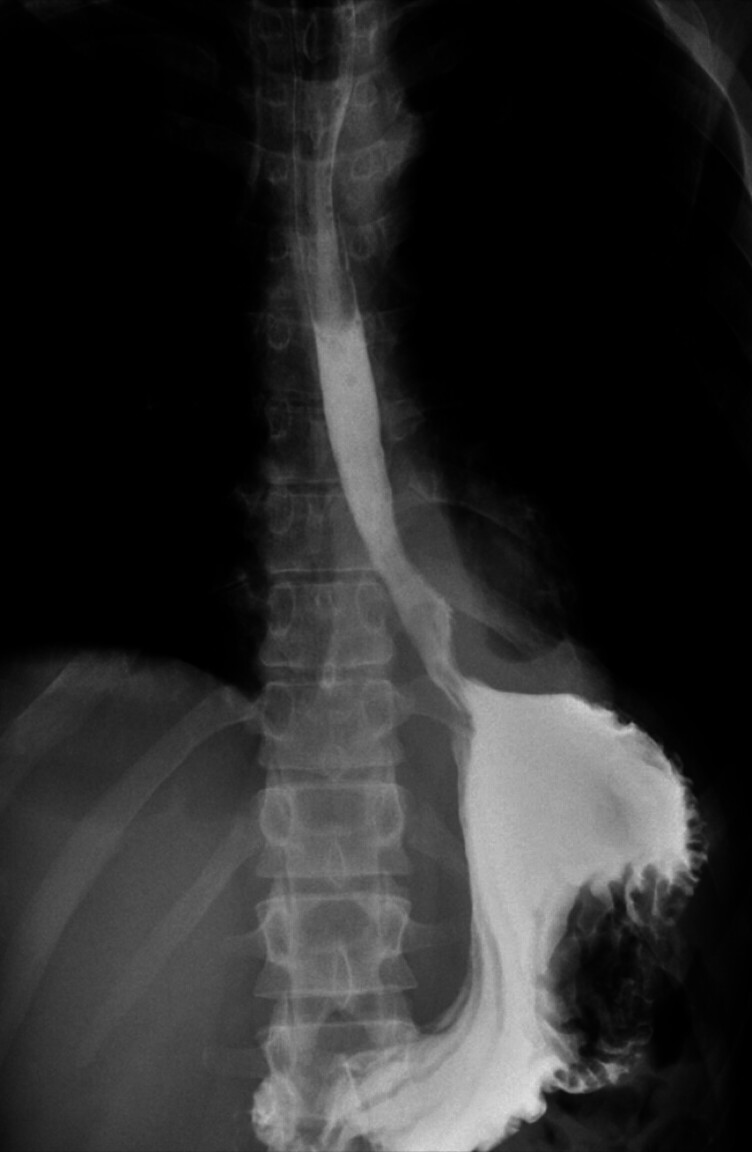
X-ray with oral contrast agent.

**Fig. 5:**
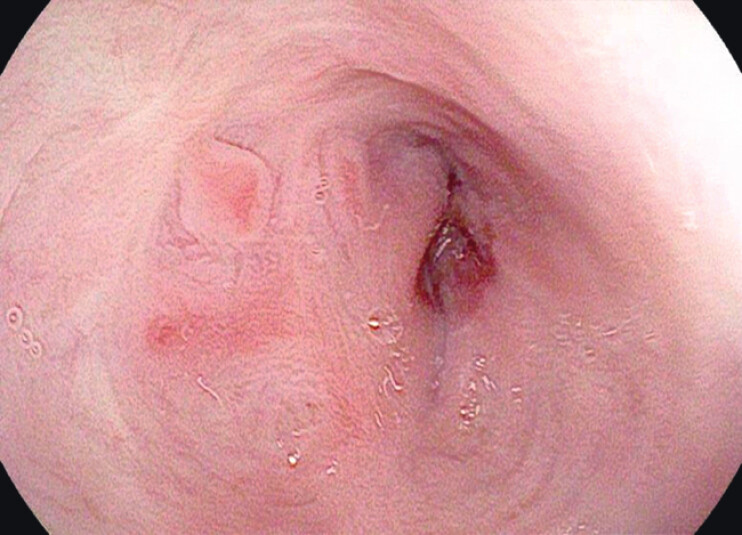
Endoscopic view of esophagus after 6 months.

When employing multidisciplinary patient care, this new device can be considered a safe therapeutic option for the successful management of esophageal traumatic perforation and not only for post-surgical complications where the data are already more established 3 .

Endoscopy_UCTN_Code_TTT_1AO_2AI

## Notes

Endoscopy E-Videos https://eref.thieme.de/e-videos
*E-Videos* is an open access online section of the journal *Endoscopy* , reporting on interesting cases and new techniques in gastroenterological endoscopy. All papers include a high-quality video and are published with a Creative Commons CC-BY license. Endoscopy E-Videos qualify for HINARI discounts and waivers and eligibility is automatically checked during the submission process. We grant 100% waivers to articles whose corresponding authors are based in Group A countries and 50% waivers to those who are based in Group B countries as classified by Research4Life (see: https://www.research4life.org/access/eligibility/ ). This section has its own submission website at https://mc.manuscriptcentral.com/e-videos .
